# The challenge of antibiotic selection in prosthetic joint infections due to *Corynebacterium striatum*: a case report

**DOI:** 10.1186/s12879-022-07270-0

**Published:** 2022-03-26

**Authors:** Amber C. Streifel, Cara D. Varley, YoungYoon Ham, Monica K. Sikka, James S. Lewis

**Affiliations:** 1 Department of Pharmacy, Oregon Health and Science University, 3181 SW Sam Jackson Park Road, Portland, OR USA; 2 Department of Medicine, Division of Infectious Diseases, Oregon Health and Science University, 3181 SW Sam Jackson Park Road, Portland, OR USA; 3 School of Public Health, Oregon Health and Science University-Portland State University, 3181 SW Sam Jackson Park Road, Portland, OR USA

**Keywords:** Antimicrobial resistance, Prosthetic joint infection, *Corynebacterium striatum*, Daptomycin

## Abstract

**Background:**

*Corynebacterium striatum* is a gram-positive facultative anaerobe found in the environment and human flora that has historically been considered a contaminant. More recently, *Corynebacterium striatum* has been implicated in human infections, including respiratory infections, endocarditis, and bone and joint infections, particularly those involving hardware or implanted devices.

**Case presentation:**

A 65-year-old man presented for washout of his left total knee arthroplasty following a revision 20 days prior. The patient underwent debridement of his left total knee and revision of the left total femur arthroplasty. Daptomycin was initiated empirically due to a previous rash from vancomycin. Operative tissue cultures grew *Staphylococcus haemolyticus*, *Staphylococcus epidermidis* and *Corynebacterium striatum*. Given concern for daptomycin resistance and the reliability of vancomycin susceptibility, daptomycin was discontinued and vancomycin initiated following a graded challenge. Within a few days, the patient developed a diffuse, blanching, erythematous, maculopapular rash and daptomycin was restarted. Over the next 72 h, his rash progressed and he met criteria for drug reaction with eosinophilia and systemic symptoms (DRESS) syndrome. Daptomycin was stopped and oral linezolid initiated; rash improved. *C. striatum* returned with susceptibility to gentamicin, linezolid, vancomycin and daptomycin. Due to concern for adverse effects on long-term linezolid, daptomycin was restarted and was tolerated for 20 days, at which point purulent drainage from incision increased. The patient underwent another arthroplasty revision and washout. Operative cultures from this surgery were again positive for *C. striatum*. Repeat *C. striatum* susceptibilities revealed resistance to daptomycin but retained susceptibility to linezolid. Daptomycin was again changed to linezolid. He completed six weeks of linezolid followed by linezolid 600 mg daily for suppression and ultimately opted for disarticulation.

**Conclusions:**

*C. striatum* has historically been regarded as a contaminant, particularly when grown in tissue culture in the setting of prosthetic joint infection. Based on the available literature and susceptibility patterns, the most appropriate first-line therapy is vancomycin or linezolid. Treatment with daptomycin should be avoided, even when isolates appear susceptible, due to the risk of development of high-level resistance (MIC > 256 µg/mL) and clinical failure.

## Background

*C. striatum* is a gram-positive facultative anaerobe found in the environment and in human nasopharynx and skin. Non-diphtheriae *Corynebacterium* species have historically been considered a contaminant. More recently, *C. striatum* has been increasingly implicated in a number of infections, including respiratory infections, endocarditis, and bone and joint infections, particularly those involving hardware or implanted devices [1]. While the specific virulence factors contributing to these infections are not well described, *C. striatum* has been noted to form biofilms on prosthetic material and has been implicated in nosocomial outbreaks. Adherence to a variety of prosthetic surfaces, including both hydrophobic and hydrophilic surfaces has been noted via binding of fibrinogen to the surface of the organism [2, 3]. Increasingly common reporting of these infections may be a result of evolving identification technologies in clinical microbiology laboratories, such as matrix-assisted laser desorption ionization-time of flight mass spectrometry (MALDI-TOF MS). Although identification of *C. striatum* has not been clinically validated on the Vitek MS (bioMérieux) or the Bruker Biotyper, the organism is available in the current Knowledge Base. MALDI-TOF MS has been reported to more accurately identify *Corynebacterium* species compared to conventional biochemical methods [4–6]. In a comparison of the Bruker Biotyper and the Vitek MS, 85 *C. striatum* isolates were identified with 100% agreement, however, 4 isolates were misclassified by the RapID CB Plus (ThermoFisher) phenotypic identification system [7].

The presence of *C. striatum* in bone and joint infections, including those with prosthetic material, remains puzzling. A recent review of *Corynebacterium* species causing orthopedic infections included 13 cases of *C. striatum*. The study concluded that *Corynebacterium* species are most often a contaminant, as defined by Infectious Disease Society of America Prosthetic Joint Infection guidelines as being detected in only 1 of at least 2 samples cultured [8, 9]. However, another retrospective review of *C. striatum* reported that when isolated from hardware, the organism was considered pathogenic in 87% of cases, as determined by the treating infectious disease physician. These patients were treated with a significantly longer duration of intravenous (IV) antibiotics compared to patients with hardware infections caused by coagulase-negative *staphylococci* [10].

## Case presentation

**Fig. 1:**
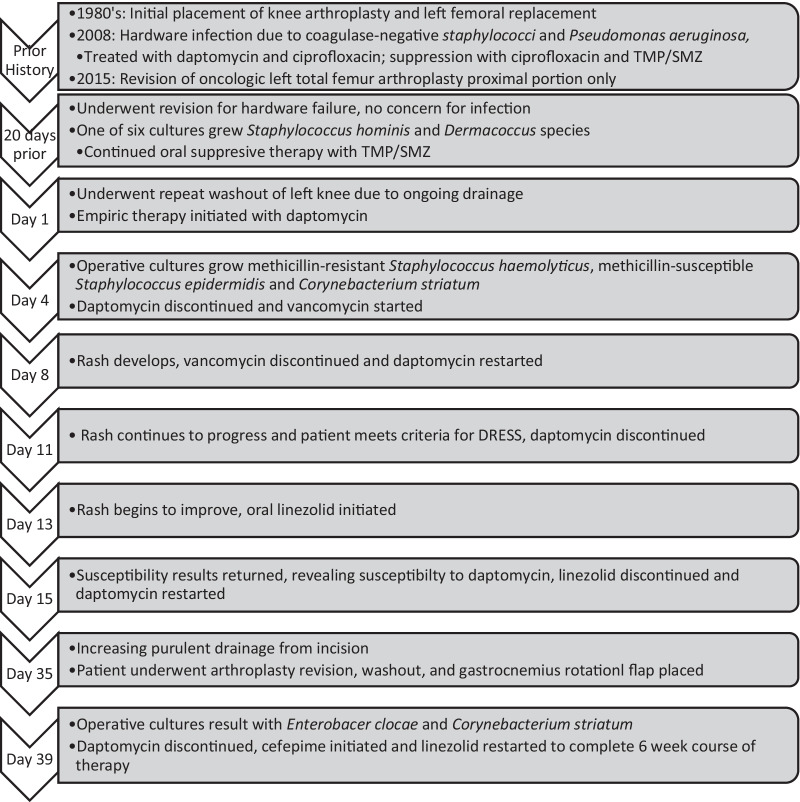
Timeline of case events. *TMP/SMZ* sulfamethoxazole-trimethoprim, *DRESS* drug rash with eosinophilia and systemic symptoms

A 65-year-old man presented for repeat washout of his left total knee arthroplasty due to ongoing drainage following revision 20 days prior. The patient’s past medical history was significant for anxiety treated with sertraline. He had undergone multiple previous revisions of his knee arthroplasty and left femoral replacement, initially placed in the 1980s (Fig. 1). In 2008, he developed hardware infections with coagulase-negative *staphylococci* and *Pseudomonas aeruginosa,* treated with daptomycin and ciprofloxacin, followed by suppression with ciprofloxacin and trimethoprim-sulfamethoxazole. His labs were only notable for mild normocytic anemia.

During his most recent revision of the tibial component and hinge mechanism for hardware failure 20 days prior to this admission, operative reports noted no concern for infection and pathology was negative for acute inflammation. One of six intra-operative cultures grew *Staphylococcus hominis* and *Dermacoccus* species; the patient continued oral trimethoprim-sulfamethoxazole suppressive therapy but discontinued oral ciprofloxacin based on cultures and concern for toxicity with long-term use.

During this admission, debridement of his left total knee wound and further revision of the left total femur arthroplasty revealed severe metallosis and a large hematoma. Operative tissue cultures were positive for methicillin-resistant *Staphylococcus haemolyticus*, methicillin-susceptible *Staphylococcus epidermidis* and *Corynebacterium striatum* via MALDI-TOF MS*,* in six of six cultures obtained. Daptomycin 6 mg/kg every 24 h was initiated empirically due to a previous rash from vancomycin. Linezolid was avoided at this time due to an anticipated treatment duration of more than 3 weeks and concern for subsequent adverse effects with prolonged linezolid therapy. Given concern for daptomycin resistance and the reliability of vancomycin susceptibility, daptomycin was discontinued and a vancomycin graded challenge was performed and tolerated on post-operative day four.

On day 4 of vancomycin, the patient developed a diffuse, blanching, erythematous, maculopapular rash and daptomycin was restarted. Over the next 72 h, his rash progressed, he became febrile to 38.9 $^\circ \mathrm{C}$, developed peripheral eosinophilia (1.45 K/cu mm, 13.8%; ref: 0–0.50 k/cu mm, 1.0–3.0%) and mildly elevated liver enzymes (AST 51 U/L, ALT 63 U/L), meeting criteria for drug reaction with eosinophilia and systemic symptoms (DRESS) syndrome. Daptomycin was stopped. His rash began to improve and he was started on oral linezolid 600 mg every 12 h on post-operative day 13. At this time, sertraline was continued despite increased risk of serotonin syndrome, as linezolid was intended to be temporary while awaiting susceptibilities. *C. striatum* susceptibilities returned on post-operative day 15 and revealed susceptibility to gentamicin, linezolid, vancomycin and daptomycin (MIC 0.064 µg/mL via E-test, breakpoint < 1 µg/mL per CLSI m-45) [11] and resistance to ceftriaxone, clindamycin, doxycycline, meropenem, and trimethoprim-sulfamethoxazole; while no interpretation was available, levofloxacin had an MIC > 8 µg/mL. The patient was re-started on daptomycin.

The patient tolerated daptomycin for 20 days but then noted increasing purulent drainage from his incision. He underwent another arthroplasty revision, washout and gastrocnemius rotational flap. One operative tissue culture from this surgery was positive for *Enterobacter cloacae* complex, and five of six operative tissue cultures were again positive for rare *C. striatum*. Antibiotics were changed to linezolid and cefepime. Cefepime was selected due to concern for AmpC induction with the use of third generation cephalosporins over long courses of antibiotic therapy in *Enterobacter cloacae*. Due to the risk of serotonin syndrome when sertraline is combined with linezolid and the patient reporting little benefit from sertraline therapy, a taper was initiated. Repeat *C. striatum* susceptibilities revealed resistance to daptomycin (reported as MIC > 1 µg/mL), via E-test but retained susceptibility to linezolid (MIC 0.5 µg/mL). He completed six weeks of linezolid and cefepime followed by linezolid 600 mg daily for suppression, due to the presence of retained hardware. The linezolid suppression was continued for just under a year and a half (522 days) until the patient ultimately opted for disarticulation. At that time the patient noted symptoms consistent with optic neuritis and peripheral neuropathy, so linezolid was discontinued. The patient was treated post-operatively for 6 weeks with oral doxycycline as repeat cultures grew *C. striatum* susceptible to doxycycline and has not re-presented with infection after 12 months.

## Discussion and conclusion

*C. striatum* exhibits limited susceptibility compared to other *Corynebacterium* species. Resistance to penicillins, cephalosporins, (including variable MICs reported for ceftaroline), carbapenems, clindamycin, and fluoroquinolones is common. Vancomycin, linezolid, and daptomycin often test susceptible [7]. Current CLSI guidelines provide interpretive criteria for susceptibility testing via broth microdilution. Daptomycin susceptibility is often determined via E-test. Disk diffusion has not been recommended since 2005, due to indeterminable differences in diffusion zone size as a result of slow diffusion because of the high molecular weight of daptomycin [11]. However, as with other organisms such as *Staphylococcus aureus* and *Enterococcus spp*, E-tests result in variability of susceptibility results depending on the calcium concentration of the Mueller–Hinton agar used [12], although calcium-supplemented E-tests are available and result in similarly accurate susceptibility testing [13]. A recently published report of 12 cases of monomicrobial *C. striatum* bone and joint infections reports treating 8 of those patients with an amoxicillin-rifampin combination. Amoxicillin MICs for 9 organisms in this series ranged from 0.38 to 3 mg/L (1 resistant organism, according to non-species related EUCAST criteria) and rifampin MICs ranged from < 0.002 to ≥ 32 mg/L (2 resistant organisms). Treatment with this combination resulted in clinical cure in 4 of 8 patients, although the majority of patients received glycopeptide therapy of unclear duration before receiving the amoxicillin-rifampin combination [14]. Application of these results in clinical practice is challenging because organisms included in this study had significantly higher β-lactam susceptibility rates than those reported elsewhere. The variability of antimicrobial agents tested and reported across the available literature makes drawing conclusions about susceptibility rates for *C. striatum* difficult. A recent systematic review of antimicrobial therapy for *C. striatum* infections reported 100% susceptibility for included isolates to vancomycin, linezolid, piperacillin/tazobactam, amoxicillin/clavulanate, and cefuroxime. However, the review included a total of 85 individual cases from the literature and reported susceptibilities for only 8 organisms for piperacillin/tazobactam, 3 organisms for amoxicillin/clavulanate, and 2 organisms for cefuroxime [15]. Reports on *C. striatum* not included in this review indicate that resistance to these agents may be higher, such as in the above study by Noussair and colleagues [14] that reported a resistance rate of 66% for piperacillin/tazobactam. These β-lactam/β-lactamase inhibitor combinations present a reasonable option for treatment if susceptibility to these agents has been confirmed.

The development of daptomycin resistance while on therapy is a significant concern, as illustrated in our case. Development of high level daptomycin resistance (MIC > 256 µg/mL) has been reported in vitro in isolates exposed to daptomycin for 24 h [12]. This has also been reported in prolonged treatment, one in a case of native valve endocarditis and in three cases of an infected left ventricular assist device [16–18]. While higher doses (> 6 mg/kg) of daptomycin have been studied for the treatment of *S. aureus* [19], the ideal dose for *C. striatum* PJI has not been established. Development of high level daptomycin resistance while on therapy has been reported in patients receiving doses ranging from 6 to 8 mg/kg [14–16, 20]. Two case reports of successful treatment of *C. striatum* endocarditis with daptomycin are available in the current literature, one that used 10 mg/kg of daptomycin and the other used daptomycin 6 mg/kg plus oral rifampin 300 mg twice daily [21, 22]. While these cases resulted in positive outcomes, more data are needed to recommend the use of high-dose daptomycin as monotherapy or with the addition of rifampin. The mechanism of daptomycin resistance in *C. striatum* is unique compared to other gram-positive organisms. In *S. aureus*, resistance to daptomycin develops via a variety of mechanisms attributed to a number of single nucleotide polymorphisms (SNPs). Acquisition of multiple SNPs results in resistance via membrane depolarization, cell wall thickening, and reduced affinity for daptomycin binding [23]. The mechanism of high-level daptomycin resistance in *C. striatum* differs in that a single mutation results in loss of function at phosphatidylglycerol synthase (pgsA2). Daptomycin activity is dependent on the bacterial cell membrane phosphatidylglycerol (PG) concentration. The pgsA2 mutation in *C. striatum* allows removal of PG from the cell membrane, altering membrane composition to maintain viability of the bacterial cell while conferring resistance to daptomycin [24]. This resistance phenotype has been shown to persist despite removal of daptomycin in culture media, unlike *S. aureus*, which often reverts once selective pressure is removed [17]. In general, routine susceptibility testing of daptomycin against *C. striatum* should not be recommended given the significant risk of development of high-level resistance on therapy.

Given our experience with this complicated case, we reviewed a series of prosthetic joint infections (PJI) with *C. striatum* isolated from operative cultures at our institution. We identified an additional 11 cases between July 1, 2015 and July 19, 2019 with a median follow-up of 1.4 years (range 0.4–4.4 years) following *C. striatum* isolation (Table 1).

**Table 1:** Additional *C. striatum* hardware infection case details (n = 11)

Age, median (range)	70.4 years (54.3–91.6 years)
Gender, female	6 (55%)
Involved joint	
Hip	5 (45.5%)
Knee	3 (27.3%)
Ankle	1 (9.1%)
Elbow	1 (9.1%)
Spine	1 (9.1%)
Prosthetic joint age, median (range)	4.5 years (0.07–38.2 years)
Prior surgical procedures, median (range)	2 (1–18)
Presenting symptoms	
Drainage	8 (72.7%)
Worsening pain	8 (72.7%)
Sinus tract formation	4 (36.4%)
Erythema	3 (27.3%)
Dislocation	2 (18.2%)
Exposed hardware	1 (9.2%)
*Additional organisms isolated with C. striatum in 10 patients with polymicrobial cultures*	
Patient	
1	MRSA
2	MRSA; *P. aeruginosa*; *E. faecalis*; *Bacillus* species, not *B. anthracis*; *P. mirabilis*
3	CoNS
4	MRSA; *P. aeruginosa*
5	*E. cloacae*
6	*E. faecalis*
7	*E. faecalis*; *C. acnes*
8	MSSA; *E. faecalis*
9	MSSA; CoNS
10	MRSA; CoNS

*MRSA* methicillin-resistant *Staphylococcus aureus*, *CoNS* coagulase-negative *staphylococci*, *MSSA* methicillin-susceptible *Staphylococcus aureus*

*C. striatum* susceptibilities were performed in seven (63.6%) patients, with all isolates susceptible to vancomycin. Susceptibilities at our institution are performed at an outside laboratory, and include ceftriaxone, clindamycin, doxycycline, erythromycin, gentamicin, levofloxacin, linezolid, meropenem, penicillin, and trimethoprim-sulfamethoxazole. Vancomycin and daptomycin susceptibilities are performed via E-test, if requested. However, only one patient had additional susceptibilities requested for their *C. striatum* isolate, which was susceptible to daptomycin, linezolid and resistant to fluoroquinolones. Ten (90.9%) patients were treated with vancomycin and one with daptomycin for a median of 42 days (range 17–105 days). Six (54.5%) required additional surgeries of the involved joint, including two amputations or disarticulations, a median of 44 days (range 5–614 days) following their isolation of *C. striatum*. Only one had *C. striatum* isolated from subsequent procedures, however this patient (#3) was initially treated with daptomycin, and was changed to vancomycin therapy following repeat *C. striatum* isolation. Daptomycin susceptibilities were not performed on either isolate from this patient. These cases highlight the appearance of *C. striatum* in complicated patients with PJI, although they likely under-represent the total cases at our institution, as the practice of identifying *C. striatum* to the species level is relatively new in our clinical microbiology laboratory.

*C. striatum* has historically been regarded as a contaminant, particularly when grown in tissue culture in the setting of PJIs, which are frequently polymicrobial. This case highlights the need to consider *C. striatum* a pathogen in certain clinical contexts. The most appropriate antimicrobial therapy for *C. striatum* PJI includes vancomycin or linezolid, but β-lactams may be considered if susceptible. Treatment of these infections with daptomycin should be avoided even when isolate appears susceptible due to the risk of developing high-level resistance leading to clinical failure.

## Data Availability

The datasets generated during and/or analyzed during the current study are available from the corresponding author on reasonable request.

## Abbreviations

*C. striatum*: *Corynebacterium striatum*
DRESS: Drug rash with eosinophilia and systemic symptoms
MIC: Minimum inhibitory concentration
MALDI-TOF MS: Matrix-assisted laser desorption ionization-time of flight mass spectrometry
AST: Aspartate aminotransferase
ALT: Alanine transaminase
CLSI: Clinical and Laboratory Standards Institute
EUCAST: European Committee on Antimicrobial Susceptibility Testing
SNP: Single nucleotide polymorphisms
pgsA2: Phosphatidylglycerol synthase
PG: Phosphatidylglycerol
*S. aureus*: *Staphylococcus aureus*
PJI: Prosthetic joint infection
